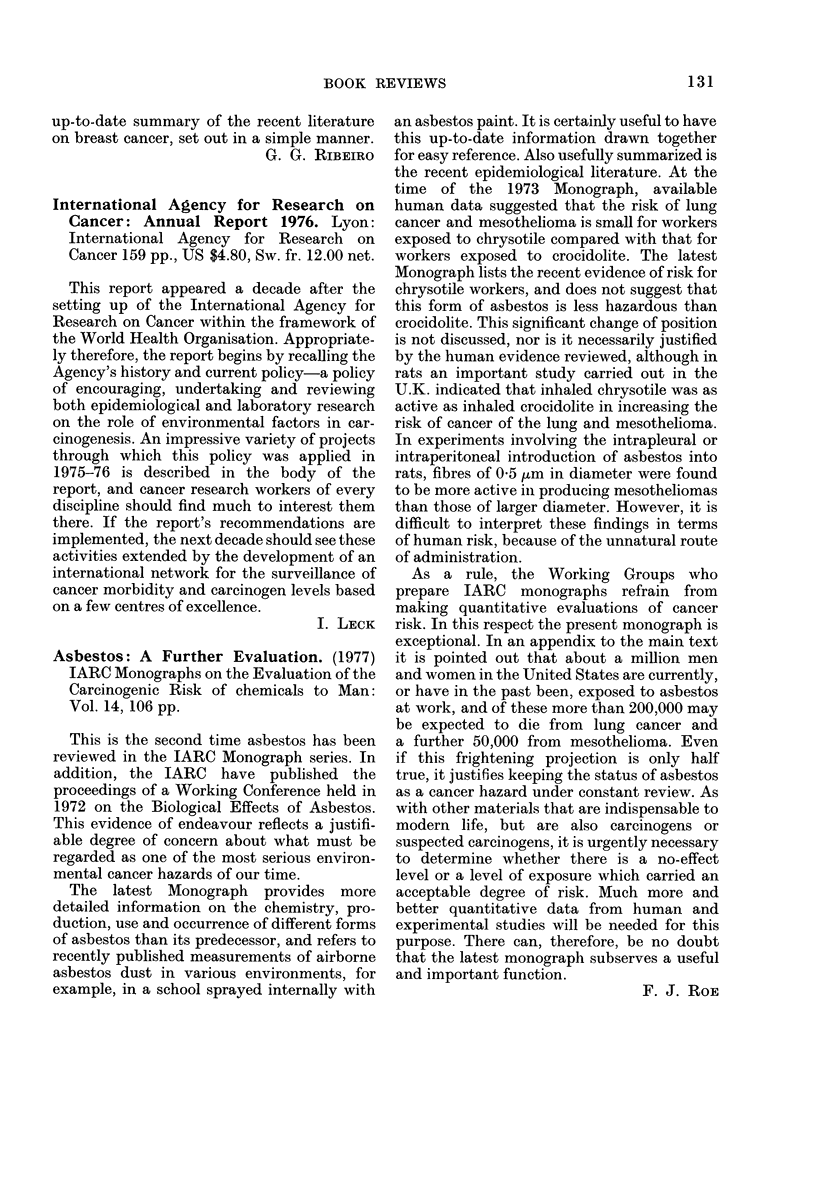# Asbestos: A Further Evaluation

**Published:** 1978-01

**Authors:** F. J. Roe


**Asbestos: A Further Evaluation.** (1977) IARC Monographs on the Evaluation of the Carcinogenic Risk of chemicals to Man: Vol. 14, 106 pp.

This is the second time asbestos has been reviewed in the IARC Monograph series. In addition, the IARC have published the proceedings of a Working Conference held in 1972 on the Biological Effects of Asbestos. This evidence of endeavour reflects a justifiable degree of concern about what must be regarded as one of the most serious environmental cancer hazards of our time.

The latest Monograph provides more detailed information on the chemistry, production, use and occurrence of different forms of asbestos than its predecessor, and refers to recently published measurements of airborne asbestos dust in various environments, for example, in a school sprayed internally with an asbestos paint. It is certainly useful to have this up-to-date information drawn together for easy reference. Also usefully summarized is the recent epidemiological literature. At the time of the 1973 Monograph, available human data suggested that the risk of lung cancer and mesothelioma is small for workers exposed to chrysotile compared with that for workers exposed to crocidolite. The latest Monograph lists the recent evidence of risk for chrysotile workers, and does not suggest that this form of asbestos is less hazardous than crocidolite. This significant change of position is not discussed, nor is it necessarily justified by the human evidence reviewed, although in rats an important study carried out in the U.K. indicated that inhaled chrysotile was as active as inhaled crocidolite in increasing the risk of cancer of the lung and mesothelioma. In experiments involving the intrapleural or intraperitoneal introduction of asbestos into rats, fibres of 0·5 $\mu$m in diameter were found to be more active in producing mesotheliomas than those of larger diameter. However, it is difficult to interpret these findings in terms of human risk, because of the unnatural route of administration.

As a rule, the Working Groups who prepare IARC monographs refrain from making quantitative evaluations of cancer risk. In this respect the present monograph is exceptional. In an appendix to the main text it is pointed out that about a million men and women in the United States are currently, or have in the past been, exposed to asbestos at work, and of these more than 200,000 may be expected to die from lung cancer and a further 50,000 from mesothelioma. Even if this frightening projection is only half true, it justifies keeping the status of asbestos as a cancer hazard under constant review. As with other materials that are indispensable to modern life, but are also carcinogens or suspected carcinogens, it is urgently necessary to determine whether there is a no-effect level or a level of exposure which carried an acceptable degree of risk. Much more and better quantitative data from human and experimental studies will be needed for this purpose. There can, therefore, be no doubt that the latest monograph subserves a useful and important function.

F. J. ROE